# Reduced cadmium(Cd) accumulation in lettuce plants by applying KMnO_4_ modified water hyacinth biochar

**DOI:** 10.1016/j.heliyon.2022.e11304

**Published:** 2022-11-04

**Authors:** Xin Yin, Yali Wang, Li'e Wei, Huajun Huang, Chunhuo Zhou, Guorong Ni

**Affiliations:** a College of Land Resources and Environment, Jiangxi Agricultural University, Nanchang, Jiangxi, 330045, China; b Key Innovation Center of Agricultural Waste Resource Utilization and Non-point Source Pollution Prevention and Control of Jiangxi Province, Nanchang, Jiangxi, 330045, China

**Keywords:** Water hyacinth biochar, KMnO4 modification, Available Cd, Soil remediation, Enzyme activities, Microbial structure and abundance

## Abstract

In this study, water hyacinth was adopted to prepare biochar followed by modification using KMnO_4_. And the modified biochars were applied in Cd contaminated soil, exploring the effects of water hyacinth biochar on lettuce growth, Cd enrichment, soil enzyme activities and microbial changes by pot experiments. Modified biochar application significantly reduced the Cd accumulation in lettuce shoots and roots. Compared to the control, the application of water hyacinth biochar at 1% rate resulted in significant reduction of Cd contents by 40.7% and 33.7% in the shoots and roots of lettuce. Also, the reduction was 33.3% and 20.8% compared with the application rate of unmodified biochar. With the increase of biochar application, the amount of Cd was absorbed by lettuce shoots and roots showing significant reduction of plant Cd accumulation in response to the biochar application rate. Additionally, the lowest available Cd concentration in soil (1.34 mg kg^−1^) was obtained with the application of modified biochar at 1% rate, which might be the main reason for the lower Cd concentration in lettuce shoot and root parts. Furthermore, structural analysis showed that Cd was fixed on the modified biochar, in a passivated state, by larger specific surface area, more active sites and more stable covalent binding complexes leading to a strong decrease of the available Cd in the soil. Moreover, it was concluded that the increment of the enzyme activities in the soil was up to 2.51 times significantly following the application of modified water hyacinth biochar with 3% amount. Lastly, 16sRNA sequencing showed that biochar addition may lead to changes of microbial structure and abundance in soil.

## 1 Introduction

Recently, the accumulation of heavy metals such as cadmium (Cd), nickel, arsenic and mercury in soil showed an increasing tendency, due to mining, metal smelting, irrigation water pollution and atmospheric subsidence (Shen et al., 2018; Wang et al., 2021). Heavy metals can be accumulated by crops grown in highly contaminated soils, then entering the food chain, which ultimately poses a serious threat to human health. For instance, long-term exposure to Cd caused “Itai Itai disease” (Kasuya et al., 2011). Moreover, the latest research shows that even low levels of Cd exposure have been associated with increased incidence of cardiovascular and cerebrovascular diseases such as atherosclerosis, hypertension and diabetes, while high dose exposure may lead to the outbreak of a variety of cancers (Tinkov et al., 2018; Soderholm et al., 2020; Xu et al., 2021). Therefore, it is necessary to take effective remediation measures to reduce the heavy metal content of crops grown on contaminated soil.

Actually, using biochar as adsorbent to treat heavy metal contaminated soils had been proved to be an effective method (Nie et al., 2018; Zhang et al., 2020a, Zhang et al., 2020b). Compared with other contaminated soil remediation ways, biochar passivation has been recently recognized as an efficient implement without secondary pollution (Xiang et al., 2021). Studies have shown that heavy metals in soil can be complexed and adsorbed on the internal and external surfaces of biochar by metal ions with different functions. However, the initial biochar (unmodified biochar) products present limited adsorption capacity, due to their limited surface area, pore characteristics and functional groups. Thus, in order to expand the scope of practical application, it is necessary to increase the active site, specific surface area and other physical and chemical properties of biochar by modifications (Liu et al., 2020; Zhang et al., 2020a, Zhang et al., 2020b; Huang et al., 2021; Masrura et al., 2021). Many modification approaches have been reported, including immobilization of active metal ions, acid-base treatment, physical removal impurities and chemical oxidation (Ahmed et al., 2014). However, it should be emphasized that the selection of modification methods was highly dependent on the types of the pollutants.

Actually, some oxidants could increase the contents of oxygen-containing functional groups on the biochar (Enaime et al., 2020). KMnO_4_ is an effective biochar modification reagent, providing MnO_2_ in the modified biochar (Feng et al., 2014). Compared with initial biochar, MnO_2_ modification has a higher specific surface area with lower isoelectric point, which can greatly improve the adsorption performance for trace metals (Zhang et al., 2020a, 2020b). Wang et al. (2015) studied the adsorption capacities of heavy metals by KMnO_4_ treated hickory wood biochar. Studies have proved that the biochar modified with KMnO_4_ has a larger specific surface area loaded with a number of oxygen-containing functional groups and MnOx ultrafine particles (Qiu et al., 2019; Fan et al., 2018). However, the application of KMnO_4_ modified biochar to heavy metal adsorption in wastewater has been reported frequently, but a little KMnO_4_ modified biochar applied in heavy metal contaminated soil.

Our previous experimental results showed that water hyacinth biochar application significantly reduced the Cd accumulation by 73.6% and 38.1% in lettuce shoots and roots, respectively. However, to get better adsorption efficiency of water hyacinth biochar, the time of the pyrolyze process should be prolonged which results in higher additional costs (Zhang et al., 2016; Yan et al., 2017; Liu et al., 2020). Hence, it is practicable to optimize the pore structure of water hyacinth biochar by modification methods. It was speculated that the combination KMnO_4_ for water hyacinth biochar modification can influence the pore structure, functional groups, element/minerals composition, carbon defects of biochar. However, the application of KMnO_4_ modified water hyacinth biochar to Cd-contaminated soil has not been reported.

In this study, water hyacinth was adopted to prepare biochar followed by modification using KMnO_4_. And the modified biochars were applied in Cd contaminated soil, exploring the effects of water hyacinth biochar on lettuce growth and Cd enrichment. Three main contents could be investigated:(1) determine the effects of water hyacinth biochar modified by KMnO_4_ on lettuce growth and heavy metal accumulation within the plant; (2) explore the impact of biochar application rate on the redistribution of Cd in various geochemical fractions; (3) evaluate the changes in enzyme and microbial activity induced by biochar in the Cd-contaminated soil.

## 2 Materials and methods

### 2.1 Preparation of water hyacinth biochar

First, the dried water hyacinth were pyrolyzed at 300 °C for 0.5 h to obtain the pristine biochar by the method described in Zhou et al. (2017). For KMnO_4_ modification, the pristine biochar was first mixed with 100 mL KMnO_4_ (0.5 g.L^−1^) and stirred for 24 h. Then, the biochar/KMnO_4_ mixtures was put into an oven at 80 °C for drying. Last, the mixture was prepared by heating at 300 °C for 1 h in a muffle furnace under the protection of N_2_ atmosphere. The resulting pyrolyzed products were washed with 0.5 mol.L^−1^ HCl to remove the impurities, then washed repeatedly with pure water until pH of ∼7.0 and dried at 60 °C. Finally, all biochars were treated by ball milling to obtain the uniform samples.

### 2.2 Experimental design

The soil samples were crushed and screened through 10 IS which laid flat on plastic film after sundries were removed. The exact amount of Cd applied to the studied soil to make the Cd concentration in the soil reach 5 mg kg^−1^. Then, the soil was kept stable for 8 weeks by stirring to ensure that the Cd was evenly mixed in the soil. 10, 20, 40 and 60 g water hyacinth biochar was added to the 2 kg Cd contaminated soil (The mass ratio of 0.5%, 1%, 2% and 3%) putting into plastic flowerpot which kept the soil in the flooded state (about 3 cm water layer), each dealing with 3 replications. Finally, 5 lettuce seeds were sown in each pot and set 2 plants in each pot after maintaining soil water content at 60% of soil field capacity. All potted plants are placed in the artificial greenhouse to avoid the rain erosion but not to control other natural conditions.

### 2.3 Biochar characterization

Surface morphology of the samples were determined using scanning electron microscopy (SEM) (JEOL JSM-6400, Japan) equipped with an energy dispersive X-ray fluorescence spectroscopy (EDS, Oxford Instruments Link ISIS) for analyzing surface elements. The functional groups on the surface of the adsorbent were analyzed by IRPrestige-21 transform infrared spectrometer (Thermo Fisher Scientific, Amenica). The surface organic functional groups were determined by Fourier transform infrared (FTIR) spectroscopy (Nicolette is 50, Thermo Fourier, USA).

### 2.4 Samples collection

Lettuce shoots were harvested after 40-days growth. First, the collected lettuce plants placed in a foam box filled with dry ice in the dark and transported back to the laboratory as soon as possible after removed the large soil. The yield of lettuce in each flowerpot was measured by weighing all of the plant materials, including leaf blades and petioles (without roots). Plants were separated into roots and shoots after transporting samples to the laboratory. The plant roots were washed with sterile water after shaking the plant roots to remove the loose soil in the roots in a sterile working environment. The cleaned turbid fluid was centrifuged (12,000 rad/s, 10 min). The solids was collected and stored in the refrigerator at −80 °C for microbiological analysis. Then, the cleaned shoots and roots fresh weight was measured after drained with a filter paper. Lastly, the samples were first dehydrated in an oven at 105 °C for 30 min and further dried to a constant weight at 60 °C. The dried samples were ground to powder and sieved to pass an 80-mesh sieve for chemical analysis.

### 2.5 Laboratory analyses

Total Cd detection of the plant, roots and soil were analyzed by atomic absorption spectrophotometry (ICE 3300, Perkin Elmer, USA). Available Cd concentrations were measured according to the Standard Methods (APHA, 2002). Soil urease and catalase activities were measured using Solarbio activity detection kit. Urease activity was defined that 1 μgNH_3_-N per g soil sample per day as an enzyme activity unit (U/g.soil). Catalytic degradation of 1 μmol H_2_O_2_ per g of air-dried soil sample per day was defined as a catalase enzyme activity unit (U/g.soil). The surface morphology of corroded surface of each specimen was examined by Scanning electron microscope (SEM) of TESCAN VEGA3. The chemical compositions were analyzed by energy dispersive X-ray spectroscopy (EDS) of AZtec X-MaxN80.

### 2.6 Analysis of soil microbial community abundance

The samples were sent to Novogene Company for 16sRNA sequencing. A polymerase chain reaction (PCR) targeting 16S rRNA genes was performed using the forward primer 347F (5′-CCTACGGRRBGCASCAGKVRVGAAT-3′) and the reverse primer 802R (5′-GGACTACNVGGGTWTCTAATCC-3′) for bacteria. The final data were obtained through a series of processing and comparative analysis with the database. Operational taxonomic unit (OUT) is a Numerical taxonomy method. By clustering the Marker gene sequences in 16S sequencing, the taxon can reflect the attribute characteristics of species. Generally, the threshold of 97% similarity is set as 1 OTU. OTUs clustering and species classification analysis were carried out to obtain the analysis results of each sample. Then, on the premise of all the analysis results, the diversity of species was analyzed to find out the differences among the different samples.

### 2.7 Statistical analysis

Statistical analyses were performed using the SPSS 18.0 statistical package program (SPSS Institute, USA). One-way analysis of variance (ANOVA) and Turkey's test were used to assess the statistical differences between the treatments. The level of significance was set at P < 0.05.

## 3 Results and discussion

### 3.1 Effect of biochar treatments on Cd uptake by lettuce shoots

The effects of water hyacinth biochar addition with the amount of 1% rate on Cd uptake in lettuce shoots before and after modification in Cd contaminated soil were explored. The main reason of the application amount at 1% rate was that the excessive application of biochar may lead to the decrease of lettuce yield. With the increase of biochar application rate within a certain range (0–1%), lettuce yield showed an upward trend. Figure S1A depicted that the peak fresh weight of lettuce with the application amount at 1% rate was 18.10 g. Many studies have reported that cadmic stress could disrupt photosynthetic efficiency, nutrients uptake, biomass production, and crops yield in Cd-contaminated conditions (Andresen and Küpper., 2013; Carstensen et al., 2018). There is no doubt that the application of biochar would alleviate Cd stress due to the decline of available Cd in soil to increase the yield of lettuce grown in cadmium-contaminated soil. In addition, the nutritive properties of biochar and its application to improve soil physical and biological properties may also be key factors in increasing lettuce yield. However, with the increasing use of modified biochar, the fresh weight of lettuce showed decreased tendency significantly. The minimal value of the fresh weight of lettuce was only 12.7 g with the biochar application of 3% amount, which was 29.8% lower than that of 1% rate. Some studies suggest that excessive biochar application may also lead to a decrease in the availability of nitrogen, phosphorus and other trace elements in the soil (Griffin et al., 2017; Liu et al., 2018). It may be the main reason for the yield reduction of lettuce caused by excessive biochar. Additionally, compared with the initial water hyacinth biochar, the yield of lettuces was significantly increased by 21.5% after modification, as described in Figure S1B.

**Figure 1:**
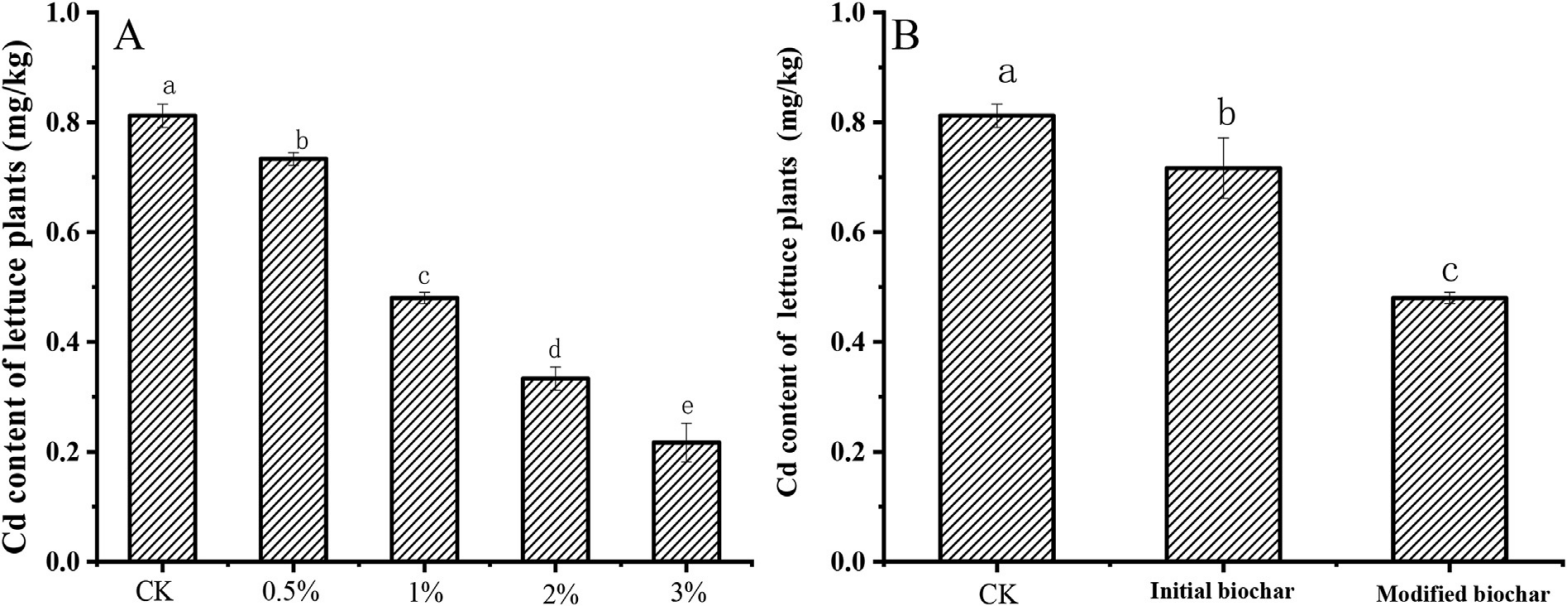
Comparison of the Cd content of lettuce plant after water hyacinth biochar application. A, Cd contents of after initial and modified biochar application; B, Cd contents of modified biochar by different amounts.

When modified biochar was applied, the Cd content of lettuce plant was significantly lower. The peak value was only 0.48 mg kg^−1^, which was not only 40.7% and 33.3% lower than that of the control and the biochar before modification. And, the more biochar was applied, the less Cd content was absorbed by lettuce. Figure 1A shows that the Cd content of lettuce shoots was only 0.22 mg kg^−1^ when the biochar modified by KMnO_4_ amount was 3%, only 27.2% of the control and 56.3% lower than that of 1% rate application. As shown in Figure 1B, the Cd content of lettuce shoots was 0.72 mg kg^−1^ with water hyacinth biochar application of 1% rate on day 40, which decreased by 11.1% compared with 0.81 mg kg^−1^ of the control. It is reasonable to speculate that KMnO_4_ modification may enhance the adsorption capacity of biochar for Cd in soil, reducing Cd transfer to the plant (discussion later).

### 3.2 Effect of biochar treatments on Cd uptake by lettuce roots

**Figure 2:**
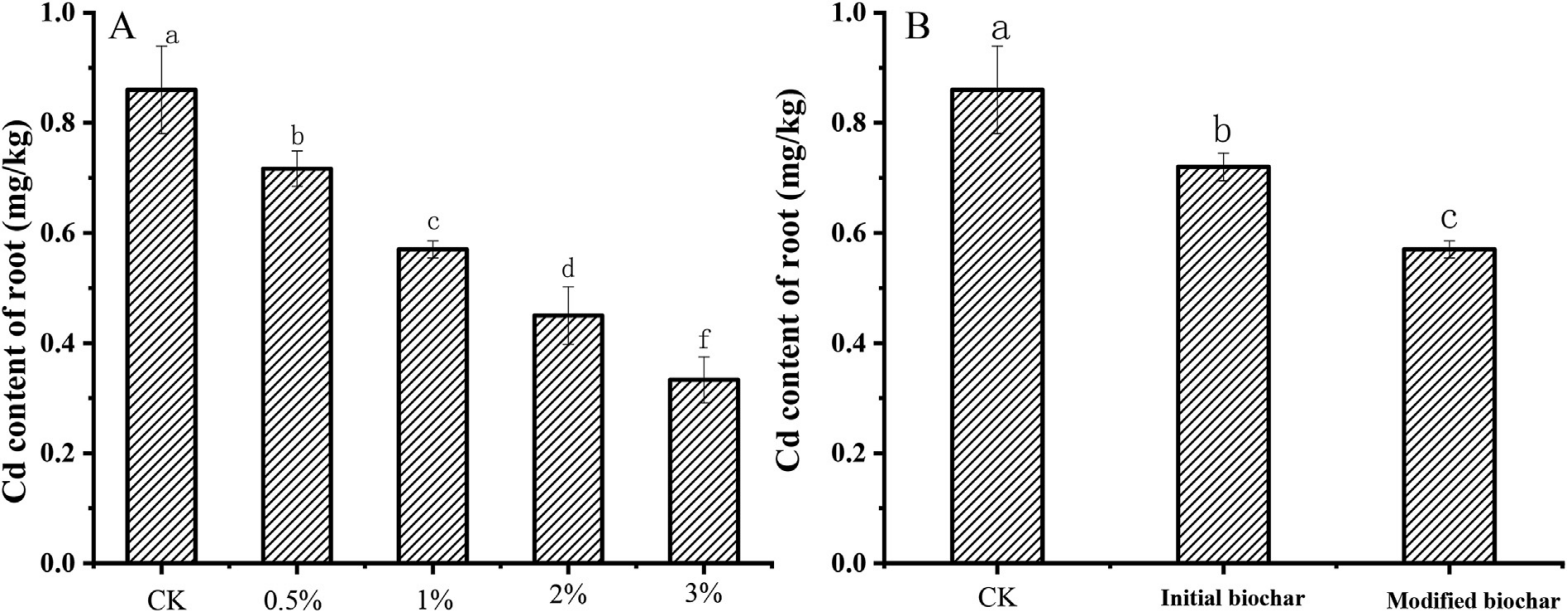
Comparison of the Cd content of lettuce roots after water hyacinth biochar application. A, Cd contents of modified biochar by different amounts; B, Cd contents of after initial and modified biochar application.

The effects of different kinds of water hyacinth biochar on the absorption of Cd in lettuce roots were also investigated. Similar to the absorption of Cd in lettuce shoots, the uptake of Cd in lettuce roots decreased with biochar application. After applying modified biochar of 0.5% rate, the content of Cd in lettuce roots was 0.72 mg/kg, which was 16.3% lower than that of control at 0.86 mg kg^−1^. When the application amount of modified biochar increased to 1%, 2% and 3%, the Cd content of lettuce roots decreased gradually, which was only 0.57, 0.45 and 0.33 mg kg^−1^, respectively. The minimum value was only 38.4% of the control, As Shown in Figure 2A. The Cd uptake of lettuce roots decreased distinctly with the increase of biochar application. By comparing the Cd content of lettuce root with and without modification water hyacinth biochar application, it was found that KMnO_4_ modification significantly reduced the transfer of Cd from soil to lettuce roots (Figure 2B). Compared with the Cd of the lettuce roots after the initial biochar application by 1% amount, the value by modified biochar addition with the same amount decreased 20.8% at the 40th day of growth. Regardless of heavy metal deposition in the atmosphere, the reduction of Cd absorption in lettuce roots will inevitably result in less Cd transfer to the plant, ultimately leading to the decrease of Cd content in edible parts of lettuce.

### 3.3 Effect of biochar application on available Cd concentration of soil

**Figure 3:**
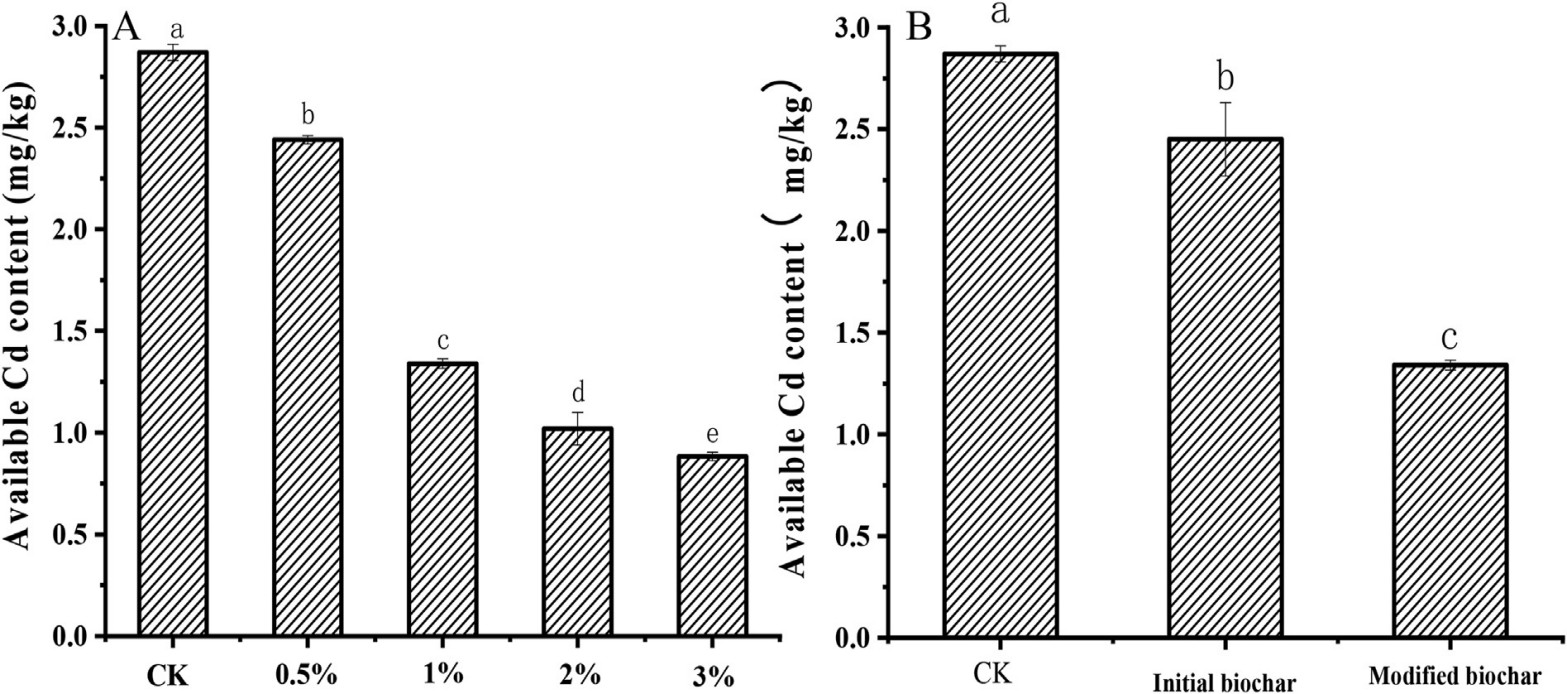
Comparison of the available Cd content of soil. A, available contents of modified biochar by different amounts; B, available Cd contents of after initial and modified biochar application.

Many studies have shown that the available Cd in soil can explain its mobility and biological toxicity effects accurately (Ayoub et al., 2003; Kulikowska et al., 2015). In order to explore how water hyacinth biochar affects the Cd absorption of the lettuce in the soil, the available Cd concentration in the soil was determined at the end of the experiment. As shown in Figure 3A, with the increase in the amount of water hyacinth biochar, the available Cd in the soil showed a significant downward trend. The content of available Cd in the soil with 0.5% amount of modified biochar at 2.44 mg kg^−1^ was 15.0% less than that of the control. The available Cd in the soil will decrease with the amount of modified biochar increasing. When the application dose of biochar was increased to 3%, the available Cd in the soil decreased to 0.88 mg/kg, which was 69.3% and 63.9% less than that of the control and the treatments of 0.5%, respectively. Moreover, in order to distinguish the difference of biochar properties before and after modification significantly, initial biochar was not prepared under the optimal conditions in this study. As shown in Figure 3B, compared to the control, the available Cd in the soil was only reduced from 2.87 mg kg^−1^ to 2.45 mg kg^−1^ after the application of 1% rate initial biochar. The value decreased by 16.4%. The adsorption efficiency of water hyacinth biochar was greatly improved by KMnO_4_ modification. After applying 1% rate modified biochar, available Cd in soil reduced to 1.34 mg kg^−1^, only 46.7% of the control and 54.7% of the initial biochar.

### 3.4 Comparison of surface structure of biochar before and after application

**Figure 4:**
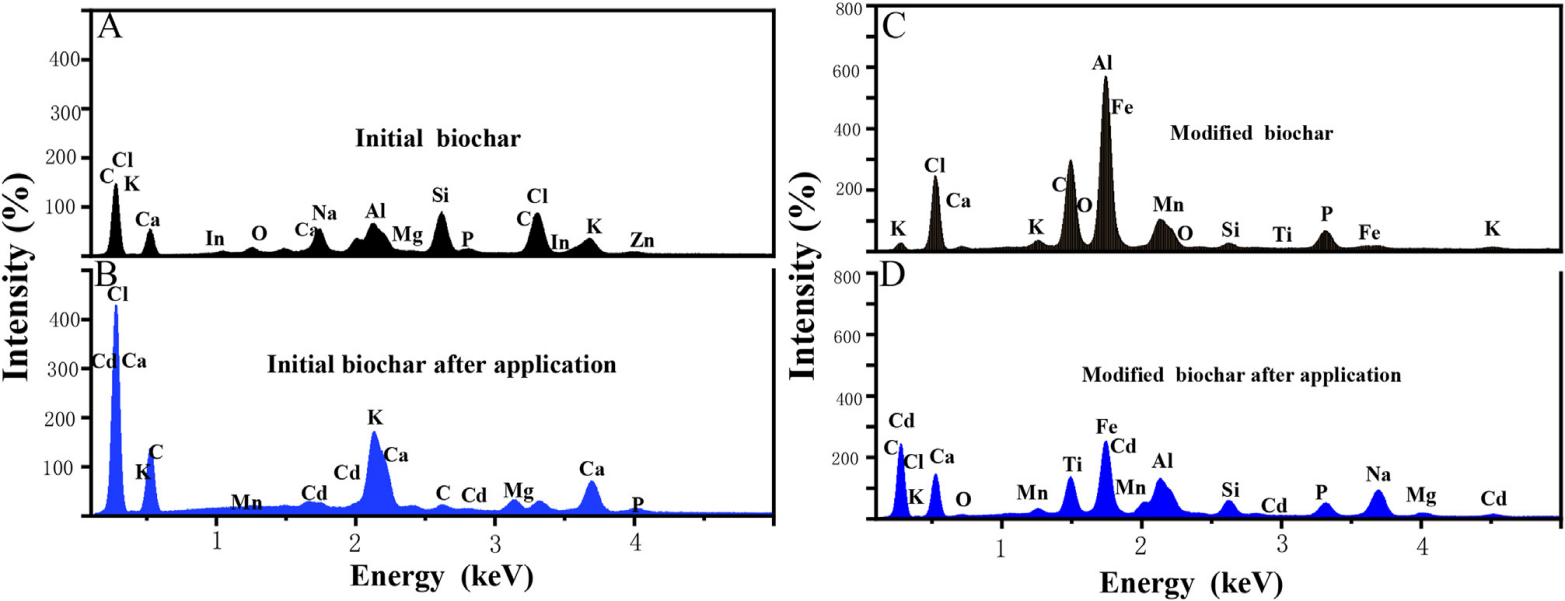
EDS analysis of biochar before and after modification and application. A, EDS results of initial biochar; B, EDS results of initial biochar after application; C, EDS results of modified biochar; D, EDS results of modified biochar after application.

As shown in Figure S2-A, the surface of water hyacinth biochar prepared initially is smooth with neat structure. It indicated that the carbonization didn't cause serious damage to the structure. It was found that there were many kinds of elements such as Cl, Al and Si on the surface of the water hyacinth biochar initially prepared by EDS analysis, but no Cd element by the description in Figure 4A. However, Figure S2-B depicted that the surface structure of the biochar changed due to the decomposition of the biochar appearing some holes after mixing and full contact with the soil. In addition, many aggregates adhered to the surface of water hyacinth biochar after application. EDS analysis of the aggregates by Figure 4B showed that there were high abundance of Cd elements, as well as Fe, Mn and other metal elements in the aggregates.

It could be found from Figure S2-C that the surface structure of KMnO_4_ modified biochar became loose and rough. Meanwhile, many small pores were distributed in the loose large pores. Xia et al. (2015) supposed that the loose large pores meant a more complex spatial structure providing more active sites. The results of Table 1 proved that the surface area, pore volume and pore diameter increased by KMnO_4_ modification. EDS analysis of Figure 4C depicted that there was a lot of element Mn on the surface of the biochar. Due to the strong oxidation of the biochar by KMnO_4_, the content of carbon decreases and the content of oxygen increases in the modified biochar, just as Wang et al. (2015) described it. Undoubtedly, it was demonstrated a large amount of manganese oxide particles attached to the biochar during the modification process. Figure S2-D showed more aggregates forming many holes and granulated bumps were adhered on the modified biochar compared with Figure S2-B. As mentioned earlier, Mn–O bonds tend to form more stable Mn–O–Cd complexes with Cd elements. As expected, EDS results found that the Cd energy spectrum of the aggregates taken from the modified biochar was much higher than that of control after applied to soil, compare Figure 4B and D.

**Table 1:** Specific surface area and pore structure parameters of biochar before and after modification.

Samples	Surface area (m^2^·g^−1^)	Pore volume (cm^3^·g^−1^)	Pore diameter (nm)
Initial biochar	0.935	0.008	31.2
Modified biochar	1.17	0.012	39.1

### 3.5 FTIR analysis before and after application

**Figure 5:**
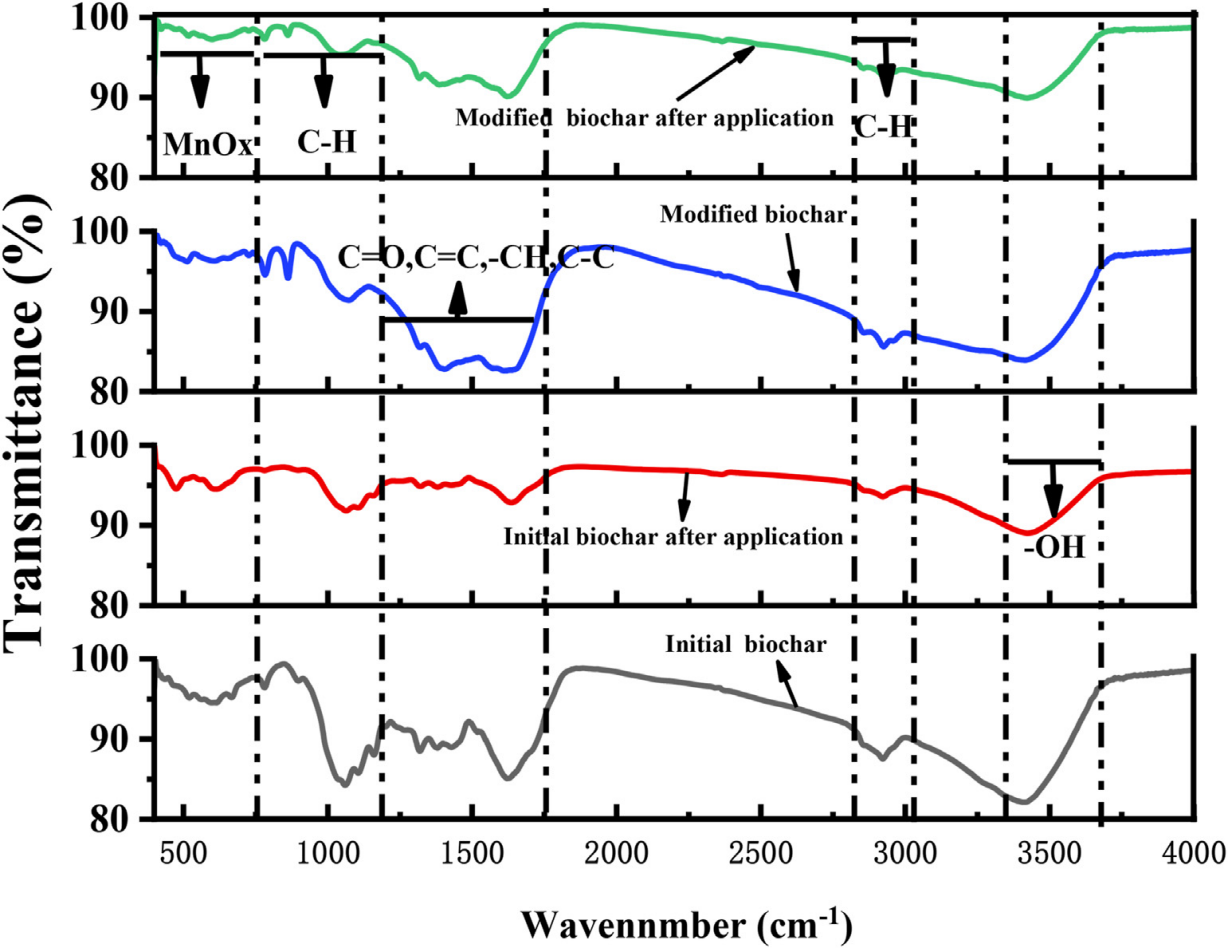
FTIR analysis of biochar before and after modification and application.

Figure 5 is the FITR analysis of biochar before and after modification and application. After adsorption, the functional groups on the surface of modified biochar barely changed, indicating that the types of functional groups didn't change during the adsorption process (Chen et al., 2015). But the Mn–O tensile vibration of modified biochar at the wavelength of 555 cm^−1^ demonstrated that the modified manganese oxide has been successfully loaded onto the surface of biochar. After modification, the stretching vibration of –OH enhanced at 3440 cm^−1^ and shifted to 3448 cm^−1^ (Liu et al., 2018). It showed that the loading of manganese oxide can increase –OH groups on the surface of biochar, which could provide more adsorption sites for modified biochar in the adsorption process. Also, there is evidence that the –OH group is involved in the adsorption process, because its –OH tensile vibration at 3448 cm^−1^ reduced to 3444 cm^−1^ (Tan et al., 2018). Therefore, KMnO_4_ modification enhanced the complexation of –OH functional groups on biochar surface to Cd in the soil. Additionally, the C]O functional groups of modified biochar at 1708 cm^−1^ shifted to 1698 cm^−1^ after applied to soil, which may be related to the metal-π electron interaction between the unloaded manganese oxides of biochar and metal cations (including Cd) in soil during the adsorption process (Tan et al., 2018). However, the wave number of Mn–O functional groups decreased after the modified biochar was applied to soil, indicating that there was a chemical interaction between the loaded manganese oxides in the biochar and metal ions during the adsorption process. These results showed that manganese oxide played an important role in the adsorption of Cd by biochar applied to heavy metal contaminated soil.

### 3.6 Effect of biochar treatments on soil enzyme activities and microbial diversity

As an important component of soil, soil enzymes were the catalyst of various soil biochemical reactions and soil microbial activities closely related to the degradation and purification of soil pollutants. Its activities can reflect the intensity of soil biochemical reactions and the pollutant degradation ability (Ni et al., 2018; Subrahumnyam et al., 2016). Thus, the effects of biochar on urease activity in Cd contaminated soil are presented in Figure S3. Similar to the trend of Cd enrichment in lettuce shoots and roots, biochar application can increase soil urease activity. The activity of soil urease increased by 66.7% after applying water hyacinth biochar (256.2 mgNH_4_^+^·kg^−1^·h^−1^) compared with that of the control (153.7 mgNH_4_^+^·kg^−1^·h^−1^). Furthermore, soil urease activity increased to 313.4 mgNH_4_^+^·kg^−1^·h^−1^ after the same amount of modified biochar was applied to soil, which was 2.04-fold of that of the control, and also increased by 22.3% compared with that before modification.

**Figure 6:**
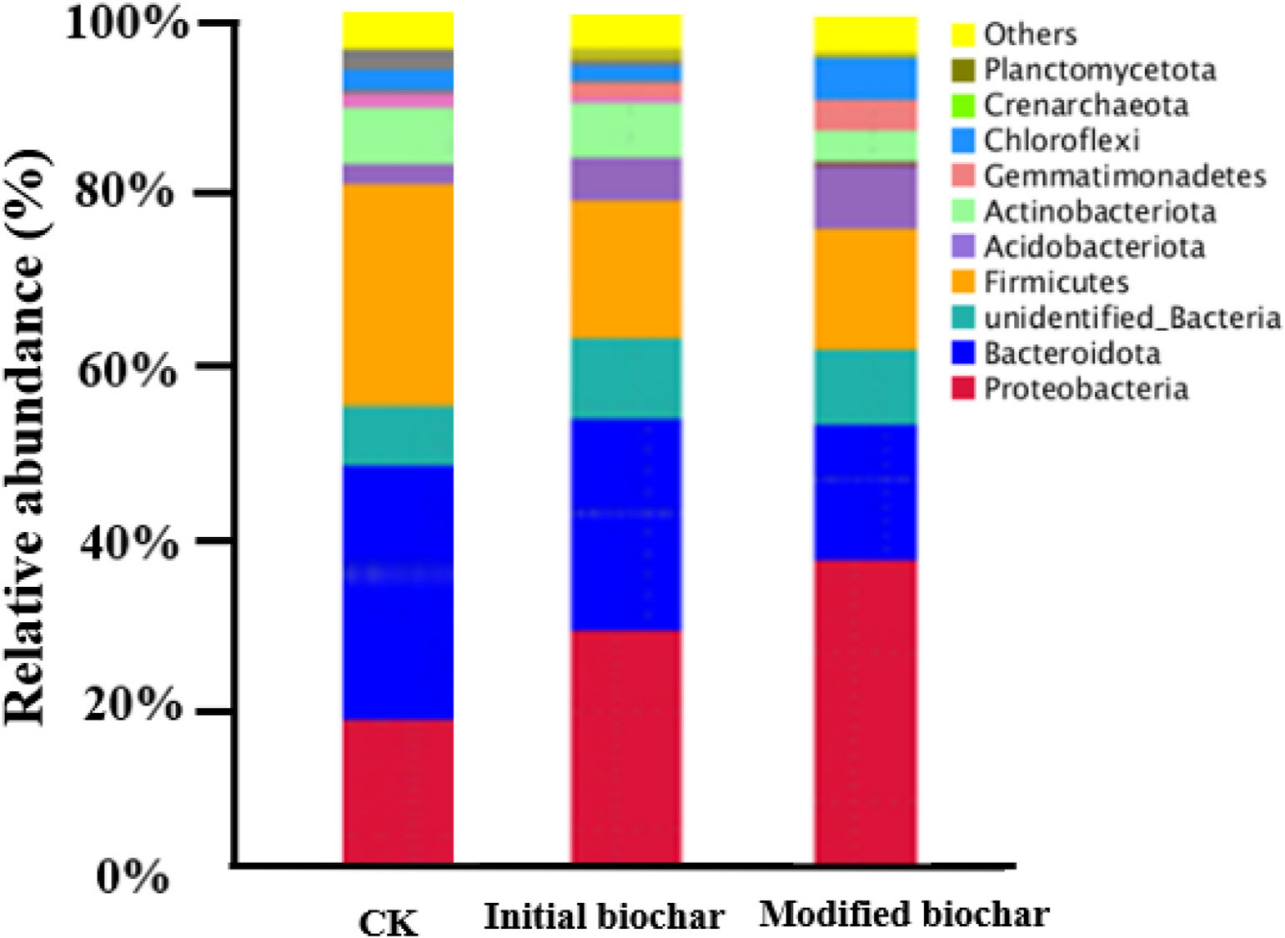
Microbial diversity analysis of biochar before and after modification and application.

Finally, the soil samples were taken and sequenced to investigate the effects of biochar application on the microbial structure and abundance of Cd contaminated soil. As shown in Figure 6, different biochar application resulted in differences in the relative abundance of dominant bacteria in Cd contaminated soil. Biochar application significantly reduced the relative abundance of *proteobacteria*. The abundance of *proteobacteria* with modified biochar application was lower than that of initial biochar and control.

## 4 Discussion

Water hyacinth is one of the most harmful aquatic plants in the world, which seriously affects the water environment and its ecosystem, having a great negative impact on water transportation and safety and flood prevention (Lu et al., 2007). Therefore, the treatment and disposal of water hyacinth has become an important topic in the field of environmental protection. Water hyacinth biochar has recently shown potential for metal adsorption from industrial, agricultural, and household wastewaters (Zhang et al., 2016). Previous studies have proved that biochar can be used as a good material for the passivation and remediation of heavy metal-contaminated soil (Yan et al., 2017; Liu et al., 2020). The surface of biochar contains abundant oxygen-containing functional groups, which can form stable metal complexes with heavy metals in soil to play a good adsorption and stabilization role. Biochar prepared with water hyacinth as raw material has a better adsorption effect on heavy metals, but it also faces the problem of excessive cost due to high energy consumption if the adsorption efficiency needs to be improved. In this study, water hyacinth was adopted to prepare biochar followed by modification using KMnO_4_. Modified biochar application significantly reduced the Cd accumulation in lettuce shoots and roots. Compared to the control, the application of water hyacinth biochar at 1% rate resulted in significant reduction of Cd contents by 40.7% and 33.7% in the shoots and roots of lettuce. Also, the reduction was 33.3% and 20.8% compared with the application rate of unmodified biochar. Also, experimental results showed that with the increase of modified biochar addition, Cd uptake in lettuce phants and available Cd content in soil showed a decreasing trend. There was a significant positive correlation between Cd content in lettuce and available Cd content in soil, indicating that modified biochar addition reduced the availability of soil cadmium, resulting in reduced uptake of cadmium by plants.

The initial biochar including water hyacinth biochar products present limited adsorption capacity, due to their limited surface area, pore characteristics and functional groups. KMnO_4_ modification also was to increase the physical and chemical properties of water hyacinth biochar such as active site and specific surface area. Previous studies have shown that small amounts of Mn, Fe and other metal elements derived from soil easily form active functional groups such as Mn–O and Fe–O on the surface of biochar, which could combine with heavy metal Cd^2+^ to form stable complexes such as Mn–O–Cd and Fe–O–Cd easily (Wang et al., 2016; Xue et al., 2019). Structural analysis by SEM, EDS and FTIR showed that Cd was fixed on the modified biochar, forming Mn–O–Cd in a passivated state, by larger specific surface area, more active sites and more stable covalent binding complexes leading to a strong decrease of the available Cd in the soil. Due to the strong effects of biochar adsorption and active functional groups after water hyacinth biochar was added to Cd contaminated soil, Cd was fixed on the biochar leading to a sharp decrease in the available Cd in the soil. Thus, lettuce roots and shoots reduce the absorption of Cd after biochar application. KMnO_4_ modification increases the specific surface area of water hyacinth biochar and attaches a large amount of Mn to form Mn–O covalent bond. When there is a lot of heavy metal Cd in the soil, it is much easier to adsorb a lot of Cd and form stable Mn–O–Cd complexes. This is the main reason why KMnO_4_ modified biochar can greatly reduce the available Cd in the soil.

Biochar application can increase the activity of soil enzymes through many aspects of regulation. First, previous studies suggested that the application of biochar itself could enhance the activity of soil enzymes. The findings were in line with Lee et al. (2011) and Cui et al. (2013) who found that changes in soil enzyme activity were related to an increase in soil pH. There's no doubt that the application of biochar can raise the pH of soil. Moreover, Oleszczuk et al. (2014) reported that biochar application could promote water retention and increase the pore structure of soil. An increase in the cation exchange capacity of soil by water hyacinth biochar had a positive effect on enzyme activity. Furthermore, biochar application could result in the increase of soil organic matter and decrease of soil nutrient loss (Chen et al., 2015). It is also an important reason for the increase of enzyme activity in soil. Particularly, previous studies suggested that heavy metals could seriously impact enzyme activity in soil, and enzyme activity indirectly reflected the capacity of contaminated soil to self-purify (Cui et al., 2013; Jia et al., 2017; Yang et al., 2017). The application of biochar especially modified biochar reduced the available Cd concentration in soil, leading to the weakening of heavy metals toxicity, which was the main reason for the increase of enzyme activity in soil. Hence, the activity of soil urease enhanced with the increase of the application amount of biochar. When the application amount of modified water hyacinth biochar reached 3%, soil urease activity reached 386.1 mgNH_4_^+^·kg^−1^·h^−1^, achieving 2.51 times higher than the control group, as depicted in Figure S3-B.

Furthermore, Meier et al. (2015) showed that biochar addition may lead to changes of microbial structure and abundance in soil, which was affected by biochar properties and application amount. Nielsen et al. (2014) proved that *proteobacteria* are more easily adapted to high heavy metals and acidic soils than other microorganisms. When biochar, especially modified biochar was applied to soil, it would reduce the toxicity of heavy metals because of high PH, eventually making other microorganisms grow in large quantities, such as *Bacteroidetes* and *Firmicutes*. Besides, *bacteroidetes* and *firmicutes* were more suitable for growing in the environment with high organic content and alkaline pH (Pan et al., 2016). Adding biochar is an effective way to increase soil organic matter, and the sustained release of biochar can reverse the degradation rate of soil organic matter to a certain extent. Additionally, the application of biochar resulted in a significant increase in *actinomycota* microorganisms. Kolton et al. (2011) showed that an increase in *actinomycetes* in general could contribute to the resistance of crops to pests and diseases. Therefore, applying biochar with better adsorption properties to contaminated soil may also play a certain role in plant resistance to pests and diseases.

## 5 Conclusions

The application of KMnO_4_ modified water hyacinth biochar with amount of 1% rate resulted in a reduction in Cd by 33.3% and 20.8% in the shoots and roots of lettuce compared with the application of unmodified biochar. The main reason was that modification allows more Cd to be fixed on the surface of the biochar, resulting in 45.3% reduction in available Cd in the soil. Moreover, the biochar treatments could potentially affect enzyme and microbial diversity in soil contaminated with heavy metals. In conclusion, biochar derived from water hyacinth, especially by chemical modification, has the potential to reduce the bioavailability of heavy metals in soil, improve the quality of soils and crops.

## Declarations

### Author contribution statement

Xin Yin: Conceived and designed the experiments; Performed the experiments; Analyzed and interpreted the data; Contributed reagents, materials, analysis tools or data; Wrote the paper.

Yali Wang: Performed the experiments; Analyzed and interpreted the data; Contributed reagents, materials, analysis tools or data.

li'e Wei: Performed the experiments; Analyzed and interpreted the data.

Huajun Huang; Chunhuo Zhou: Contributed reagents, materials, analysis tools or data.

Guorong Ni: Conceived and designed the experiments; Performed the experiments; Analyzed and interpreted the data; Contributed reagents, materials, analysis tools or data; Wrote the paper.

### Funding statement

This work was supported by the 10.13039/501100001809Natural Science Foundation of China (No. 52160002, 21707057), Natural Science Foundation of Jiangxi province (20192BAB213018).

### Data availability statement

Data included in article/supp. material/referenced in article.

### Declaration of interest's statement

The authors declare no conflict of interest.

### Additional information

No additional information is available for this paper.
